# Comparison of Functional Recovery of Manual Dexterity after Unilateral Spinal Cord Lesion or Motor Cortex Lesion in Adult Macaque Monkeys

**DOI:** 10.3389/fneur.2013.00101

**Published:** 2013-07-22

**Authors:** Florence Hoogewoud, Adjia Hamadjida, Alexander F. Wyss, Anis Mir, Martin E. Schwab, Abderraouf Belhaj-Saif, Eric M. Rouiller

**Affiliations:** ^1^Domain of Physiology, Department of Medicine, Faculty of Sciences, Fribourg Cognition Center, University of Fribourg, Fribourg, Switzerland; ^2^Novartis Pharma, Basel, Switzerland; ^3^Brain Research Institute, University of Zürich, Zürich, Switzerland; ^4^Department of Health Sciences and Technology, ETH Zürich, Zürich, Switzerland

**Keywords:** non-human primate, anti-Nogo-A antibody, reversible cortical inactivation, premotor cortex, corticospinal tract, injury of central nervous system

## Abstract

In relation to mechanisms involved in functional recovery of manual dexterity from cervical cord injury or from motor cortical injury, our goal was to determine whether the movements that characterize post-lesion functional recovery are comparable to original movement patterns or do monkeys adopt distinct strategies to compensate the deficits depending on the type of lesion? To this aim, data derived from earlier studies, using a skilled finger task (the modified Brinkman board from which pellets are retrieved from vertical or horizontal slots), in spinal cord and motor cortex injured monkeys were analyzed and compared. Twelve adult macaque monkeys were subjected to a hemi-section of the cervical cord (*n* = 6) or to a unilateral excitotoxic lesion of the hand representation in the primary motor cortex (*n* = 6). In addition, in each subgroup, one half of monkeys (*n* = 3) were treated for 30 days with a function blocking antibody against the neurite growth inhibitory protein Nogo-A, while the other half (*n* = 3) represented control animals. The motor deficits, and the extent and time course of functional recovery were assessed. For some of the parameters investigated (wrist angle for horizontal slots and movement types distribution for vertical slots after cervical injury; movement types distribution for horizontal slots after motor cortex lesion), post-lesion restoration of the original movement patterns (“true” recovery) led to a quantitatively better functional recovery. In the motor cortex lesion groups, pharmacological reversible inactivation experiments showed that the peri-lesion territory of the primary motor cortex or re-arranged, spared domain of the lesion zone, played a major role in the functional recovery, together with the ipsilesional intact premotor cortex.

## Introduction

Primates (humans and non-human) are characterized by an outstanding capability to perform fractionated finger movements, representing the exquisite behavioral attribute of manual dexterity [see e.g., Ref. (1, 2)]. The fine motor control of fingers via distal muscles of the forelimb depends largely on the corticospinal (CS) projection, originating mainly from the multiple motor cortical areas located in the frontal lobe [primary motor cortex, premotor cortex, supplementary motor area, and cingulate motor area; see e.g., Ref. (3–7)]. In primates, the CS projection comprises the so-called corticomotoneuronal (CM) connection, forming a direct projection of CS neurons in layer V onto spinal cord motoneurons. This CM system is a specialty of primates, and believed to be for the most part the anatomical support of manual dexterity [e.g., Ref. (1, 2, 8, 9)]. Indeed, a lesion affecting the CS projection system (comprising the CM system) provokes a deficit of manual dexterity. In the present study, such deficits were assessed in non-human primates (macaque monkeys), as a result of two distinct lesions affecting the CS-CM system: first, a lesion at the level of the cervical spinal cord, interrupting the CS projection near its target, the hand muscle motoneurons. Second, a lesion located at the origin of the CS-CM projection, in the hand area of the primary motor cortex, from which about 50% of the CS projection originates in the frontal lobe (3). Both types of lesion were followed by an immediate dramatic, and generally complete, loss of manual dexterity, which persisted for a few weeks. Subsequently, in both cases, there was a progressive functional recovery of manual dexterity, lasting several weeks, before reaching a plateau, representative of the final, stable post-lesion functional recovery of manual dexterity [see e.g., Ref. (10–13); see also Ref. (14–17)]. In our two models of CS-CM lesion, recovery of manual dexterity was incomplete.

Using quantitative readouts, we aimed to distinguish functional recovery, based on a restitution of the original movement pattern, as compared to compensatory movement strategies. The present, comparative analysis is based on results obtained in the “modified Brinkman board” task from monkeys used in earlier studies (10–13, 18–21). In monkeys subjected to motor cortex lesion, pharmacological reversible inactivation of distinct cortical territories was used to assess the role played by peri-lesion cortical territories, as well as the ipsilesional premotor cortex, in the motor control of fingers, and the recovery of manual dexterity.

## Materials and Methods

The present study uses behavioral data from adult macaque monkeys (10 *Macaca fascicularis* and 2 *Macaca mulatta*). Six monkeys underwent a spinal cord injury (SCI) at cervical level C7 (age 3–5 years, 4 males, 2 females); their experimental treatments are described in detail in Freund et al. (10, 11, 18). The remaining six were subjected to a unilateral lesion of the primary motor cortex (M1), affecting mostly the hand representation (age 4–5.5 years, 5 males, 1 female); described in detail in Kaeser et al. (12, 13), Bashir et al. (20), and Hamadjida et al. (21). All behavioral, surgical, electrophysiological, and pharmacological procedures were approved by the ethical committee in accordance to the Guide for the Care and Use of Laboratory Animals (ISBN 0-309-05377-3; 1996) and authorized by local (canton de Fribourg) and federal (Swiss) veterinary authorities. The experiments were covered by the following veterinary authorizations: FR 24/95/1, FR 44/92/3, FR 157/01, FR 157/03, FR 157/04, FR 157e/04, FR 156/04, FR 156/06, FR 157e/06, FR 185-08. In our former animal facility (used at the time of the present study), the monkeys were housed in rooms of 12 m^3^, forming groups of two to four monkeys free to interact with one another^1^. First thing in the morning, the animal caretaker transferred the monkeys to temporary cages on wheels, for subsequent transfer to a primate chair, in which the monkeys were moved to the laboratory to perform the behavioral session, usually lasting an hour [as illustrated in Ref. (19)]. In the animal room, the monkeys had free access to water and were not food deprived. The rewards (pellets) eaten during the behavioral tests were the first daily access to food. After completion of the behavioral session, the monkeys received additional food (fruits and cereals) in the laboratory. The monkeys’ body weight was monitored on each working day. No body weight loss>10% (criterion for interruption) occurred during the course of this study.

In addition, in each group of monkeys (SCI or cortical lesion), the six monkeys were further divided into two subgroups: three monkeys were subjected to intrathecal pump infusion of a function blocking monoclonal anti-Nogo-A antibody [see Table 1; see also Ref. (10, 11, 18) and (12), for detail], whereas the other three were either not treated (cortical lesion) or received a control-antibody (SCI), thus representing control animals. The anti-Nogo-A antibody treatment was aimed at neutralizing the neurite growth inhibitor Nogo-A, an intervention that was shown to promote functional recovery from CS tract lesion or motor cortex lesion in rats [see e.g., Ref. (22, 23–26) for review] and from CS tract lesion in monkeys (10, 11). However, while the present report does not address specifically the issue of the effect of the anti-Nogo-A antibody treatment, effects were previously reported for recovery from cervical cord lesion (10, 11, 18). Similarly, pilot data on the effect of the anti-Nogo-A antibody treatment were recently reported after motor cortex lesion (21) and will be presented more extensively elsewhere.

**Table 1:** **(A) Spinal cord lesion monkeys. (B) Motor cortex lesion monkeys**.

	Control-antibody treated monkeys			Anti-Nogo-A antibody treated monkeys*		
	Mk-S-C-HA	Mk-S-C-ST	Mk-S-C-BE	Mk-S-A-FR	Mk-S-A-MO	Mk-S-A-CO
Hemi-section extent (%)	90	63	75	56	80	85
Complete transection of the dorsolateral funiculus	Yes	Yes	Yes	Yes	Yes	Yes
Extent of dorsal column lesion (%)	72	47	31	2	74	44
Extent of CS and RS territory lesion (%)	100	87	93	73	100	100
Extent of ventral lesion (%)	60	19	38	0	5	100
Volume of the lesion (in mm^3^)	2.862	3.781	2.912	2.076	4.576	4.577
Functional recovery for vertical slots (%)	59	42	76	56	96	100
Functional recovery for horizontal slots (%)	41	0	79	52	71	100
Total functional recovery (vert. + horiz. slots) (%)	53	22	78	57	96	100

	**Untreated**			Treated with anti-Nogo-A antibody*		
	**Mk-C-C-GE**	**Mk-C-C-RO**	**Mk-C-C-BI**	**Mk-C-A-VA**	**Mk-C-A-SL**	**Mk-C-A-MO**

Surface of hand area on ICMS maps (mm^2^)	25	56	55	40	45	34
Lesioned hemisphere (ipsilesional hemisphere)	Left	Left	Left	Left	Left	Left
Total volume of lesion (mm^3^) gray matter (motor cortex + post-central gyrus)	48.7	14	20.13	20	78.2	41.8
Volume of lesion in post-central gyrus (mm^3^)	7.6	0	0	5.8	1.8	0
Volume of lesion in sub-cortical white matter (mm^3^)	0	0	0	0	130.6	0
Normalized value of lesion size with respect to surface of hand area on ICMS maps (mm^3^/mm^2^)	1.95	0.25	0.37	0.5	1.74	1.23
Functional recovery for vertical slots (%)	57	100	94	87	77	84
Functional recovery for horizontal slots (%)	11	90	36	91	77	60
Total functional recovery (vertical + horizontal) (%)	38	98	74	87	73	76

*Characteristics of lesions of the 12 monkeys involved in the movement pattern analysis, as well as functional recovery data derived from the modified Brinkman board task. Data taken from previous publications: (11–13, 21)*.

*The anti-Nogo-A antibody used is the mAb hNogo-A (human Nogo-A sequence) in the six monkeys, except in Mk-S-A-FR in which the mouse antibody mAb 11C7 was used [see (18) for characterization of the antibodies]. The monkeys Mk-S-C-ST and Mk-S-A-FR are Macaca mulatta whereas the other 10 monkeys are Macaca fascicularis.

In the present study, the names of the monkeys were coded to simplify the understanding of the results: the first two letters are identical for all monkeys (Mk) meaning “monkey.” The third letter (S or C) symbolizes the lesion type (spinal cord or motor cortex injury). The fourth letter (C or A) indicates whether the monkey has been treated with anti-Nogo-A antibody (A) or belongs to the control (C) group. The last two letters are the two first letters of the names the monkeys were given in the laboratory. The experimenters were blind to the treatment (anti-Nogo-A antibody or control-antibody) in the spinal lesion group of monkeys (other names were used initially); this was not the case for the motor cortex lesion group (anti-Nogo-A antibody treated versus untreated monkeys). The 12 monkeys considered in the present study appeared in previous reports focused on different aspects of recovery from SCI or motor cortex lesion (10–13, 18, 20, 21, 27–33), in which they can be identified by their corresponding ID name (see also Table 1).

**Figure 1:**
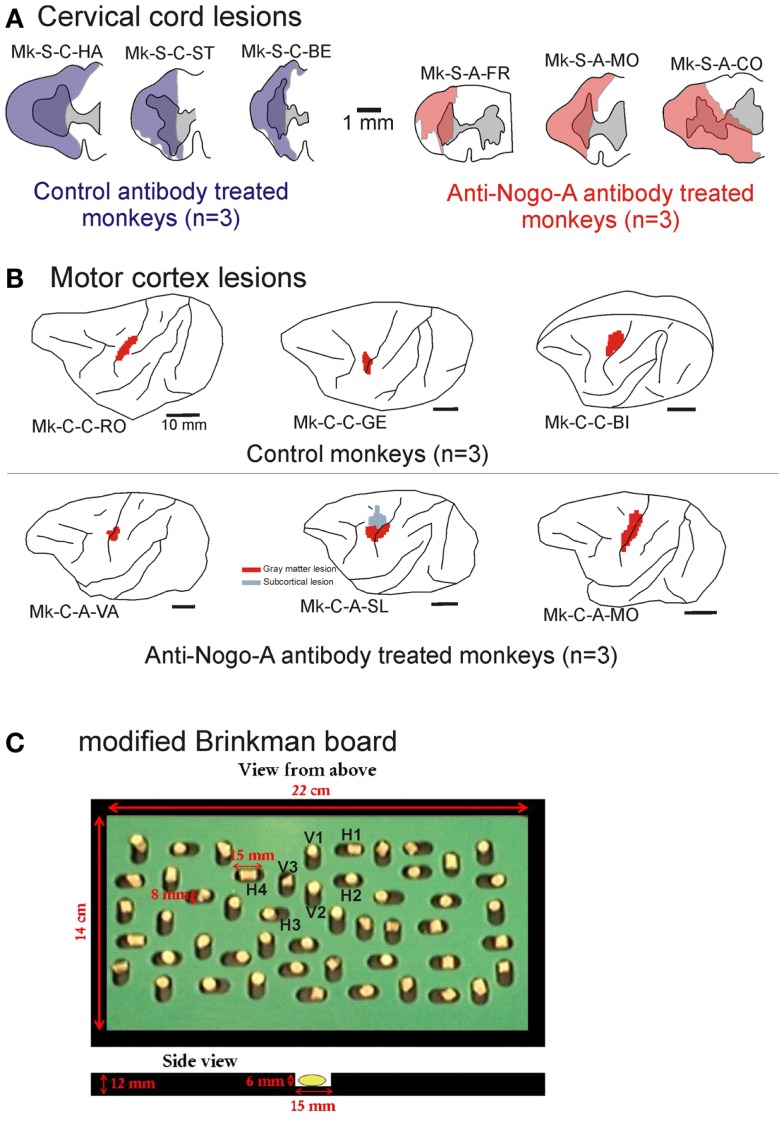
**(A)** Cross-section reconstruction in the transversal plane of the lesion position and extent (blue or red area) for the six monkeys subjected to cervical cord lesion. The gray area represents the gray matter. These lesions were illustrated in previous reports (10, 11, 18, 30, 31). **(B)** Lateral view of the left hemisphere for the six monkeys subjected to a lesion of the hand representation in M1. The red areas correspond to the lesion territory in the gray matter, as seen by transparency of the cortical surface (possible lesion territory in the white matter below the lesioned gray matter is not represented, except in Mk-C-A-SL where a lesion in the white matter, medial to the lesion in the gray matter, is also represented). These lesions were illustrated in previous reports (12, 13, 20, 21). The procedures to reconstruct the lesion and determine the volume of the cortical lesion were described in a recent report (21). **(C)** View from top of the modified Brinkman board, showing the position of the 25 horizontal and 25 vertical slots, filled with a pellet. Below, side view of an individual slot. H1–H4 are the four horizontal slots considered to assess movement types executed by the monkeys for this orientation. V1–V3 are the three vertical slots from which movement types were assessed for this orientation.

The monkeys were trained to perform a large range of manual dexterity tasks, as reported in great detail recently (19), though variable from one animal to the next over the years. However, each monkey in our laboratory during the last two decades has performed our standard test, referred to as “modified Brinkman board” task (Figure 1C), which was therefore selected for the present comparative analysis between SCI and motor cortex lesion. The monkeys were behaviorally trained and tested for a period of approximately 4 months pre- and 2–6 months post-lesion. For this purpose, the primates sat in a “primate chair” (19). This chair consists in a plexiglas box with three openings: one on the top for the head and one in front for each arm, which could be closed alternatively to assess the motor performance of each hand separately.

The modified Brinkman board task is a manual dexterity test requiring independent finger movements. The procedure used is a modified version of that introduced by Brinkman and Kuypers (34) and Brinkman (35). The modified Brinkman board (14 × 22 cm) is a perspex board pierced with 25 horizontally and 25 vertically oriented wells 15 mm long, 6 mm deep, and 8 mm wide (Figure 1C). It is placed in front of the monkey at a 40° inclination from horizontal. The task consists in retrieving small food pellets from the wells [see video sequences in Ref. (19)]. The size of the wells made it only possible to insert one finger into the well, forcing the monkey to use the precision grip (opposition of the thumb and the index finger) to grasp the pellets. The grasping of the food pellets is more challenging from the horizontal slots (precision grip combined with a pronation or supination of the wrist) than from the vertical slots (precision grip performed with the hand in its natural posture). In addition to adjustments of the wrist position, the monkeys also exhibited postural adjustments at more proximal joints (elbow, shoulder). However, as they were not visible on the video sequences focused on the hand, they were not analyzed. The behavioral data recorded in video sequences were analyzed as follows. (1) An assessment of the *score*, given by the number of pellets retrieved during the first 30 s of the task. The score was established separately for the vertical and horizontal slots, and the sum of the two yielded a total score. (2) To focus more specifically on the precision grip itself, for the pharmacological investigations (see below), a *contact time (CT)* was measured, given by the time interval (in s) from the first contact between the finger (usually index finger) and the pellet, and the moment the pellet was retrieved from the well. (3) An analysis of the type (pattern) of finger movements used for the execution of the prehension task. By analyzing *movement patterns*, we aimed to distinguish two types of recoveries: (i) “True” functional recovery where the movement patterns are conserved (re-established) in the post-lesion period, so that the same effector muscles are used to accomplish the task as before the lesion; (ii) Functional “compensation,” when the strategy to execute the task changed in the post-lesion period, recruiting other effector muscles, as compared to pre-lesion to reach the same motor goal [see e.g., (36)].

The general methodological procedures have been described in great detail in previous reports [for SCI: (10, 11, 18, 19, 27–31); for motor cortex lesion: (12, 13, 20, 21, 32, 33)]. Briefly, the temporal sequence of the experiments can be summarized as follows. Following habituation to the primate chair (2–3 months), the monkeys were first trained to execute the manual dexterity tests until reaching a plateau of motor performance, from which pre-lesion data were established during several months. In all monkeys subjected to motor cortex lesion, as well as in two out of six SCI monkeys (Mk-S-C-ST and Mk-S-A-FR), using a chronically implanted chamber above the primary motor cortex, intracortical microstimulation (ICMS) experiments were conducted to map the hand representation in the primary motor cortex. The ICMS procedure was described in detail previously [(27, 28) for SCI; (21, 32) for motor cortex lesion]. The monkeys were then subjected to either a unilateral SCI or a unilateral motor cortex lesion. The manual dexterity tests were pursued to assess the impact of the lesion (usually a total loss of the ability to perform the precision grip with the affected hand) and, then, to follow the time course of functional recovery (either spontaneous in control monkeys or supposedly enhanced in anti-Nogo-A antibody treated monkey). Finally, a post-lesion plateau of motor performance was reached after several weeks, during which post-lesion data were collected over several months. The comparison of the pre- versus post-lesion data yielded a percentage of functional recovery and allowed us to specifically address the issue of true functional recovery versus functional compensation (see above). In a later phase of the protocol, monkeys with a chronic chamber (see above) were again subjected to electrophysiological investigations (ICMS). In the case of motor cortex lesion, in order to identify which motor cortical areas may contribute to the functional recovery, pharmacological investigations were conducted, by reversibly inactivating a specific motor cortical area and assessing the impact on the manual dexterity tests. Finally, the monkeys were sacrificed under deep anesthesia for histological assessment of the cervical cord or cortical lesions [Figure 1; as previously reported: (10, 12, 18, 21, 30–32)]. The volumes of the cervical cord lesions and of the motor cortex lesions were calculated from consecutive histological sections, as previously reported in detail (11, 21).

The pharmacological investigations, consisting of reversible inactivation experiments of distinct motor cortical areas, were conducted as follows: the GABA agonist muscimol was infused at different sites in the primary motor cortex or in the premotor cortex. The reversible inactivation experiments were combined with daily behavioral sessions (three in general in each monkey), taking place usually at 1 week intervals. In a given daily session, one specific motor area was inactivated (Table 3). The daily session began with the monkey undertaking the modified Brinkman board task, successively with the contralesional hand and the ipsilesional hand, as described above. Based on the post-lesion ICMS map established a few weeks before, several sites where ICMS elicited digits’ movement were selected for muscimol infusion. For each monkey and for each motor cortical area inactivated (M1 or PMd or PMv), Table 3 indicates the number of sites where muscimol was infused (Sigma, 1 μg in 1 μl of saline 0.9% solution), using a Hamilton syringe (10 μl) inserted perpendicularly into the brain at a selected ICMS site. The volume injected at each site, the number of sites, the distance between sites and the position of the sites of infusion were determined based on the study conducted by Martin (37), on the rat brain. During an individual penetration with the syringe, muscimol was delivered at one to two depths, distant from each other by 2–3 mm (27, 38). After infusion of muscimol over several penetrations, the monkey remained quiet in its primate chair for approximately 30 min. Then, the modified Brinkman board task was repeated for each hand at three time points: 30, 45, and 60 min after offset of muscimol injections. To assess the impact of the reversible inactivation of a given motor cortical area, two parameters were measured, as described earlier: (i) the score; (ii) the CT. The CT was measured for the five first vertical slots and the five first horizontal slots visited by each hand, as previously reported (12, 13). The CT was determined by frame by frame visualization of the video sequences, corresponding to a time resolution of 40 ms (25 images/s). The score is a unique observation at each time point (30, 45, and 60 min) and therefore cannot be statistically compared with the score of reference obtained at a single time point before infusion of muscimol. In contrast, as the CT is assessed from five values for each slot orientation, statistical comparisons between the reference (on the same day before infusion of muscimol) and at each time point (30, 45, and 60 min) was undertaken using the non-parametric Mann and Whitney test. The results of the statistical comparisons are shown in Figures 8–10, with the corresponding *p* value or “n.s.” (for statistically non-significant differences with *p* > 0.05).

## Results

### Lesions

The cervical cord lesions and the motor cortex lesions are illustrated in Figure 1, for the 12 monkeys involved in the analysis of the movement patterns. Quantitative characteristics of all lesions are shown in Table 1. Representative microphotographs of the cervical cord lesion and motor cortex lesion have been shown in previous reports (11, 12, 18, 31, 39). All monkeys subjected to SCI had a complete transection of the dorsolateral funiculus and an extent of CS and rubrospinal tracts territory lesion ranging from 73 to 100%. The range of the extent of the lesion affecting the ventral funiculus was more variable (0–100%). Nevertheless, as the topic of the present study is manual dexterity, the crucial point here is that the dorsolateral funiculus was completely lesioned in all SCI monkeys (Figure 1; Table 1). As far as motor cortex lesions are concerned, in all six monkeys the hand representation in M1 was the main territory affected, as expected from infusion of ibotenic acid at ICMS sites eliciting digit movements at low threshold [see (12, 21)]. More precisely, in Mk-C-C-GE, the lesion encroached into both the pre- and post-central gyri. In the pre-central gyrus, the lesion was restricted to the M1 hand representation. In Mk-C-C-RO, Mk-C-C-BI, and Mk-C-A-MO the lesions varied in size but none encroached into the post-central gyrus. None of the above four monkeys exhibited a spread of lesion into the sub-cortical white matter. In Mk-C-A-VA, the lesion also affected mostly the M1 hand representation, with however a small spread into the post-central gyrus. The lesion of Mk-C-A-SL included an even smaller spread to the post-central gyrus, but also a large lesion of sub-cortical white matter, in addition to the targeted M1 hand area.

### Behavioral analysis

#### Score data

**Figure 2:**
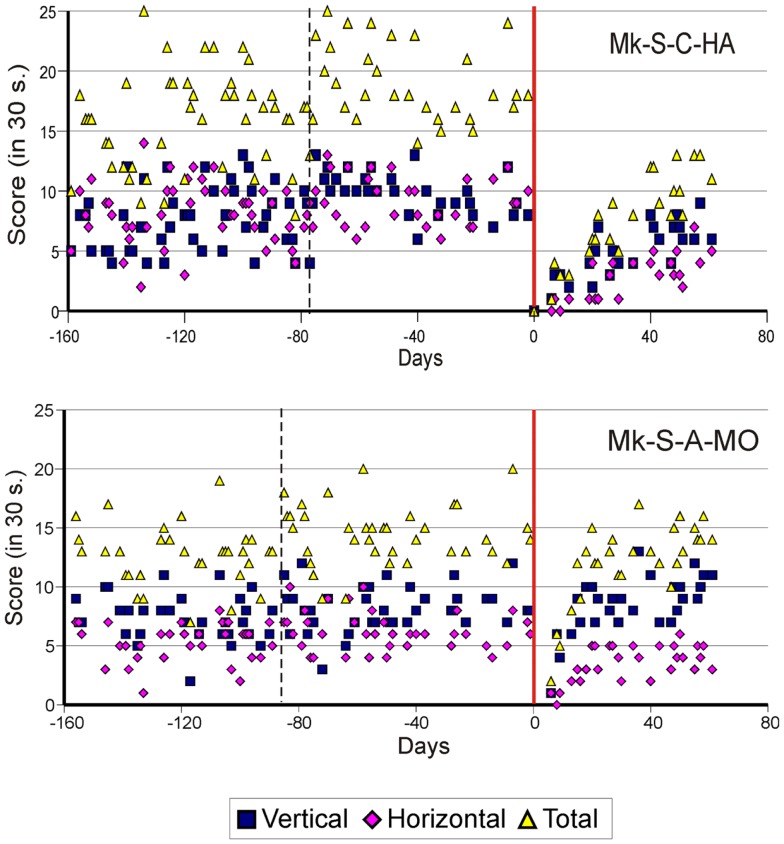
**Graphic representation of the number of pellets retrieved in 30 s pre- and post-lesion during multiple daily sessions for two representative monkeys subjected to spinal cord lesion (Mk-S-C-HA and Mk-S-A-MO)**. The red line represents the lesion date. Negative days along the abscissa are for daily sessions pre-lesion whereas positive days are for daily sessions post-lesion. The pink diamonds and the blue squares represent the number of pellets retrieved from horizontal and vertical slots, respectively. The total number of retrieved pellets in 30 s is represented by the yellow triangles. The vertical dashed line separates the initial learning phase on the left from the more stable pre-lesion plateau phase. Modified from Freund et al. (10, 11,18).

**Figure 3:**
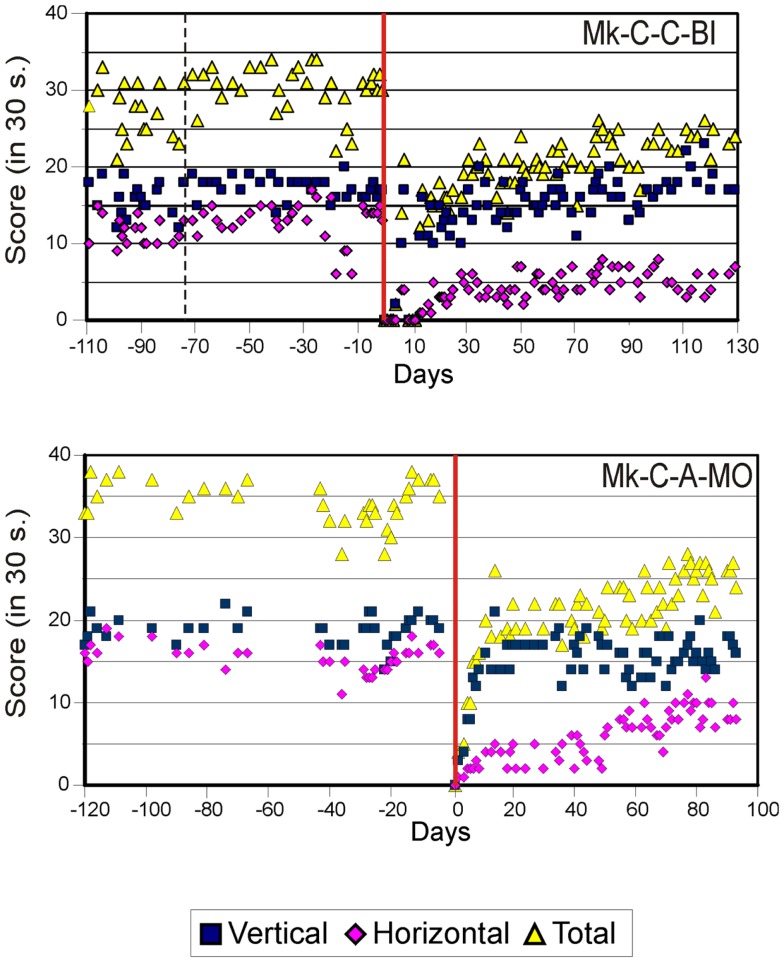
**Graphic representation of the number of pellets retrieved in 30 s pre- and post-lesion during multiple daily sessions for two representative monkeys subjected to motor cortex lesion (Mk-C-C-BI and Mk-C-A-MO)**. Same conventions as in Figure 2. Modified from Kaeser et al. (12, 13) and Bashir et al. (20).

As previously described (10–13), the score data derived from the modified Brinkman board task show that, for both types of lesion, there was a dramatic loss of manual dexterity following the injury (score dropped to zero) which persisted for up to several weeks. It was then followed by a progressive functional recovery, reaching a post-lesion plateau generally lower than the pre-lesion score after a few weeks, indicative of an incomplete functional restitution of manual dexterity. Two examples of monkeys taken from each lesion group are shown in Figures 2 and 3, respectively. When the behavioral post-lesion plateau was reached, the movement patterns produced by each monkey to perform the manual task were analyzed and compared to the pre-lesion phase.

From the score data as illustrated in Figures 2 and 3, it was possible to derive the extent of functional recovery expressed in %, obtained by dividing the post-lesion score at plateau by the pre-lesion score at plateau (Tables 1 and 2). The percentages of functional recovery will be used to confront the extent of restitution of manual dexterity with the types of movements used by the monkeys to perform the modified Brinkman board task before and after the lesion (Figures 6 and 7 below).

**Table 2:** **Statistical comparison of wrist angle (adduction) pre- versus post-lesion for horizontal slots**.

	H1	H2	H3	H4	Functional recovery (%)
**CERVICAL CORD LESION MONKEYS**					
Mk-S-C-HA	Sig. (pre > post)	Sig. (pre > post)	Sig. (pre > post)	Sig. (pre > post)	41
	*T*, *p* = < 0.001	MW, *p* = 0.009	MW, *p* = 0.005	*T*, *p* = < 0.001	
Mk-S-C-ST	Sig. (pre > post)	Sig. (pre > post)	Sig. (pre > post)	Sig. (pre > post)	0
	MW, *p* = < 0.001	MW, *p* = < 0.001	MW, *p* = < 0.001	MW, *p* = < 0.001	
Mk-S-C-BE	n.s.	n.s.	Sig.(pre < post)	n.s.	79
	MW, *p* = 0.248	*T*, *p* = 0.906	*T*, *p* = < 0.001	*T*, *p* = 0.237	
Mk-S-A-FR	Sig. (pre > post)	Sig. (pre > post)	n.s.	Sig. (pre > post)	52
	*T*, *p* = 0.004	*T*, *p* = 0.002	*T*, *p* = 0.117	*T*, *p* = 0.004	
Mk-S-A-MO	n.s.	n.s.	n.s.	Sig. (pre > post)	71
	*T*, *p* = 0.134	*T*, *p* = 0.345	*T*, *p* = 0.119	*T*, *p* = 0.021	
Mk-S-A-CO	n.s.	n.s.	Sig. (pre < post)	n.s.	100
	*T*, *p* = 0.877	*T*, *p* = 0.159	*T*, *p* = 0.006	MW, *p* = 0.328	
**MOTOR CORTEX LESION MONKEYS**					
Mk-C-C-GE	Sig. (pre > post)	Sig. (pre > post)	Sig. (pre > post)	Sig. (pre > post)	11
	(*T*, *p* = 0.005)	(MW, *p* = 0.003)	(*T*, *p* = 0.003)	(*T*, *p* = 0.027)	
Mk-C-C-RO	n.s.	n.s.	n.s.	n.s.	90
	*T*, *p* = 0.795	MW, *p* = 0.069	*T*, *p* = 0.183	*T*, *p* = 0.693	
Mk-C-C-BI	Sig. (pre < post)	Sig. (pre < post)	n.s.	Sig. (pre < post)	36
	*T*, *p* = < 0.001	*T*, *p* = < 0.001	*T*, *p* = 0.224	*T*, *p* = < 0.001	
Mk-C-A-VA	Sig. (pre < post)	n.s.	Sig. (pre < post)	Sig. (pre < post)	91
	*T*, *p* = 0.037	*T*, *p* = 0.167	*T*, *p* = 0.040	*T*, *p* = 0.020	
Mk-C-A-SL	Sig. (pre > post)	Sig. (pre > post)	Sig. (pre > post)	Sig. (pre > post)	77
	MW, *p* = < 0.001	MW, *p* = < 0.001	*T*, *p* = < 0.001	*T*, *p* = < 0.001	
Mk-C-A-MO	n.s.	Sig. (pre > post)	n.s.	n.s.	60
	*T*, *p* = 0.109	*T*, *p* = 0.009	*T*, *p* = 0.698	*T*, *p* = 0.750	

*H1–H4 refer for the four horizontal slots analyzed in the modified Brinkman board task (Figure 1C). The table shows if there was a significant difference (“Sig.”) in wrist adduction angle in the pre-lesion versus post-lesion phase and which one showed the larger angle. The second line for each monkey shows which statistical test was used (T, Student T-test, MW, Mann–Whitney) and the corresponding p value*.

#### Movement patterns

In 18 pre-lesion and 18 post-lesion daily training sessions at plateau, the movement patterns assessed for the hand affected by the lesion were precisely analyzed from video sequences to assess stereotyped movements performed by the monkeys in the modified Brinkman board task. The horizontal and the vertical slots were investigated separately, as they involved clearly distinct joint synergies. Six different movement types were identified, three for each slot orientation (horizontal and vertical), as illustrated by Movies S1–S6 in Supplementary Material (videos sequences 1–6; also accessible at the following URL address: http://www.dartfish.tv/Dispatch.aspx?target=collection&CR=p96281c64853&sh=li&aid=cfe44ff5-27f3-4c48-afaa-f23d08a82919

**Figure 4:**
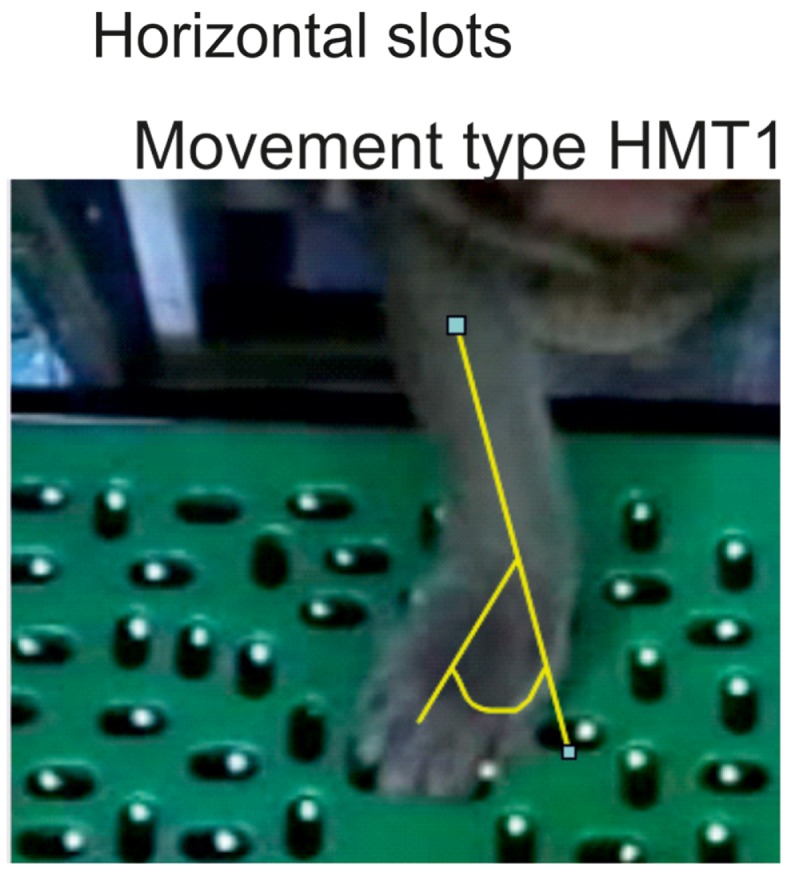
**When using the movement type 1 (HMT1) to retrieve a pellet from a horizontal slot, the wrist adduction was assessed quantitatively by measuring the angle as illustrated in yellow**.

To retrieve pellets from the horizontal wells, most monkeys used the same movement type, catching the pellet with the tips of the thumb and of the index finger (precision grip), while adducting the wrist (referred to as *horizontal movement type HMT1*). As a reminder, an adduction consists of moving a body part toward the central axis of the anatomical position (defined as a standing position with the arms alongside of the body and the palm of the hand pointing in the front). In this case, an adduction of the wrist means a movement of the wrist toward the digit V. The angle of adduction of the wrist was measured (Figure 4). To avoid bias due to the change of angle of the forearm with respect to the Brinkman board, only four wells located in the central part of the board were analyzed (slots H1–H4 in Figure 1C). Another strategy consisted of catching the pellet with the thumb and the middle phalanx of the index finger. This movement requires less wrist adduction and no precision grip (*horizontal movement type HMT2*). The third movement strategy was to approach the pellet from behind by performing an arm rotation and a flexion of the wrist and to grasp it using the precision grip, corresponding to *horizontal movement type HMT3*.

For pellet retrieval from the vertical slots, three movements types were also identified, based on three wells located in the center of the Brinkman board (Figure 1C; slots V1–V3). The *vertical movement type VMT1* corresponds to the following sequence: first reaching to the slot with the forearm; second, flexion of the fingers to grasp the pellet without simultaneous forearm movement; third, supination of the forearm to bring the reward to the mouth. In the next strategy (*vertical movement type VMT2*), after a first reaching phase with the forearm, there was a simultaneous grasping with the fingers and a forearm supination, terminated by transporting the pellet to the mouth. In the last type of movement (*vertical movement type VMT3*), the monkey also started with a reaching phase, followed by the grasping of the reward with the fingers, while the wrist made an ulnar adduction and the forearm began to supinate. With the combination of those three movements, the monkey scooped up the pellet from the slot.

##### Results from analysis of movements in SCI monkeys for horizontal slots

**Figure 5:**
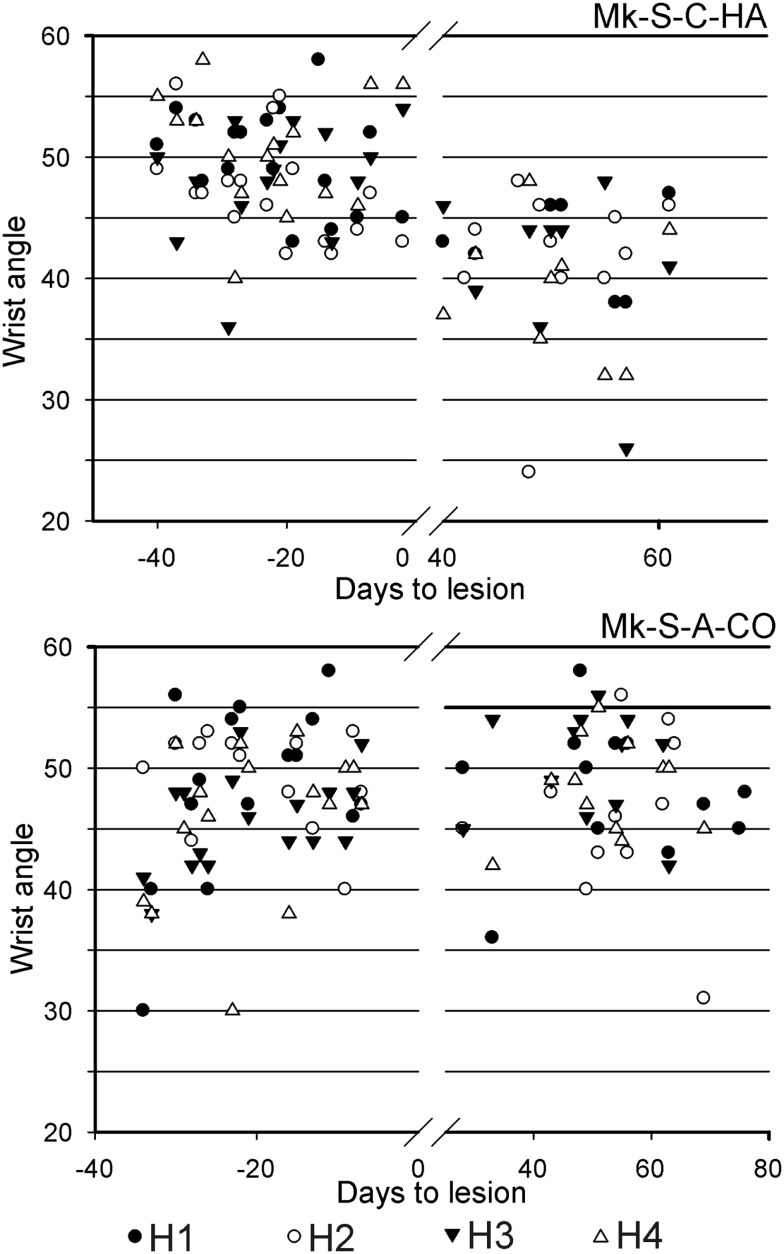
**For movement type HMT1, used to retrieve pellets from the horizontal slots, the wrist angle measured for the hand affected by a cervical cord lesion was plotted as a function of time, corresponding to a daily behavioral session, pre-lesion (negative days) or post-lesion (positive days)**. The data are presented here for two representative monkeys. On top, in Mk-S-C-HA, there was a change of wrist angle when comparing the movement pre-lesion versus post-lesion (decrease of wrist angle post-lesion). In contrast, in Mk-S-A-CO, the wrist angle was comparable pre- and post-lesion. See Table 2 for statistical analysis on all monkeys and all horizontal wells’ locations.

A significant change of wrist adduction angle to grasp pellets from horizontal slots has been observed in several monkeys subjected to spinal cord lesion (Table 2, upper part), especially prominent and systematic in Mk-S-C-HA, Mk-S-C-ST, and Mk-S-A-FR, the three monkeys with the lowest functional recovery (see top panel of Figure 5 for Mk-S-C-HA). The change consisted in a significant decrease of the wrist angle post-lesion. Note that one of these monkeys (Mk-S-C-ST) was totally unable to retrieve pellets from horizontal wells post-lesion. The third control animal with good spontaneous functional recovery (Mk-S-C-BE) did not show a decrease of wrist angle post-lesion, but rather significantly increased wrist angle for the slot H3 (Table 2, upper part). Similarly, in the anti-Nogo-A antibody treated group, two animals (Mk-S-A-MO, Mk-S-A-CO) with good functional recovery (71 and 100%) showed no change in the wrist angle in most cases (three slots out of four). The wrist angles are plotted pre- and post-lesion for Mk-S-A-CO in the bottom panel of Figure 5.

**Figure 6:**
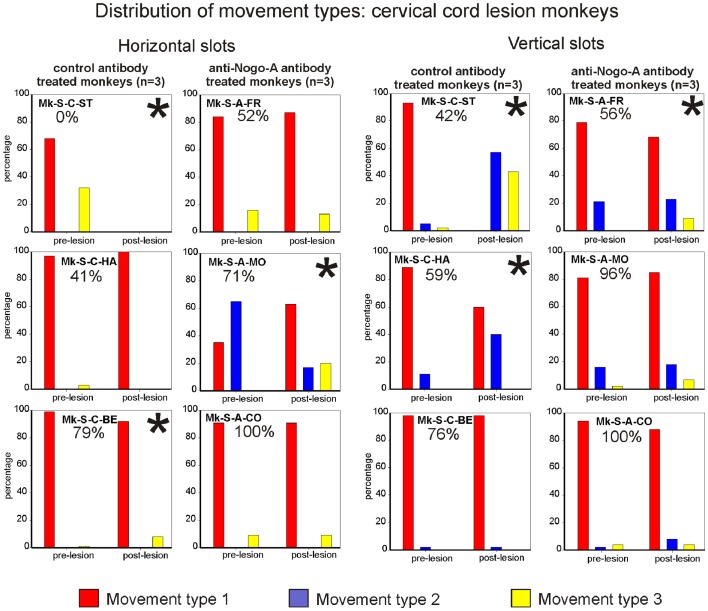
**Distribution of movement types observed for grasping pellets from the horizontal slots (left column) or from the vertical slots (right column) for the six monkeys subjected to cervical cord lesion**. *Significant change in the distribution of movement types for the execution of the task between the pre- versus post-lesion periods (chi-square test, *p* < 0.05). For Mk-S-C-ST, it has been considered that there was a significant change in movement patterns as this monkey was unable to execute the task after the lesion.

The motor strategy used to execute the modified Brinkman board task for the horizontal slots was analyzed and it turned out that five out of six monkeys preferentially used the *horizontal movement type HMT1*, both in the pre- and post-lesion periods (Figure 6, left column). Monkey Mk-S-A-MO preferentially used the *horizontal movement type HMT2* pre-lesion, and switched to *horizontal movement type HMT1* in most post-lesion trials. Two other monkeys exhibited a statistically significant change of movement types’ distribution post-lesion (Mk-S-C-BE and Mk-S-C-ST), the latter incapable of performing the grasping post-lesion. There was no correlation between significant changes of motor strategy and functional recovery (Figure 6, left column). The monkeys presenting the best recovery (Mk-S-A-CO) and the poorest recovery (Mk-S-C-HA), respectively, did not show any significant change in movement type distribution.

##### Results from analysis of movements in SCI monkeys for vertical slots

In monkeys with spinal cord lesion, the vertical slot results relating to movement type distribution are similar to the results related to wrist adduction (angle change) for horizontal slots. In other words, statistically significant changes were associated with the monkeys exhibiting the lowest functional recovery (below 60%; Figure 6, right column). The monkeys with the best functional recovery (Mk-S-A-CO, Mk-S-A-MO, Mk-S-C-BE) did not show any significant change in movement type distribution between the pre- and post-lesion phases. In all monkeys, the preferred movement to grasp pellets from vertical slots in the pre-lesion phase was *vertical movement type VMT1* (Figure 6, right column). This is the simplest of the three types of movement and is probably the most efficient. A significant decrease in the use of *vertical movement type VMT1* is correlated to a diminution of functional recovery, most impressively seen in Mk-S-C-ST.

##### Results from analysis of movements in cortical lesion monkeys for horizontal slots

In monkeys subjected to cortical lesion (see Table 2, bottom part), significant changes in wrist adduction angles were also found between the pre- and post-lesion phases. Mk-C-C-GE, a monkey that presented a poor functional recovery for horizontal slots (11%), exhibited systematic and significant decreases of wrist angles post-lesion. However, the same change has been seen in Mk-C-A-SL, a monkey with a much better recovery (77%). Nevertheless, these two monkeys exhibited the largest lesion (see Table 1). In two monkeys (Mk-C-C-BI, Mk-C-A-VA), whose functional recoveries were heterogeneous (36 and 91% respectively), there was a significant increase of wrist adduction in the post-lesion phase (Table 2, bottom part). In Mk-C-C-RO (recovery 90%) and Mk-C-A-MO (recovery 60%), no significant change has been found, except for one slot in the latter animal.

**Figure 7:**
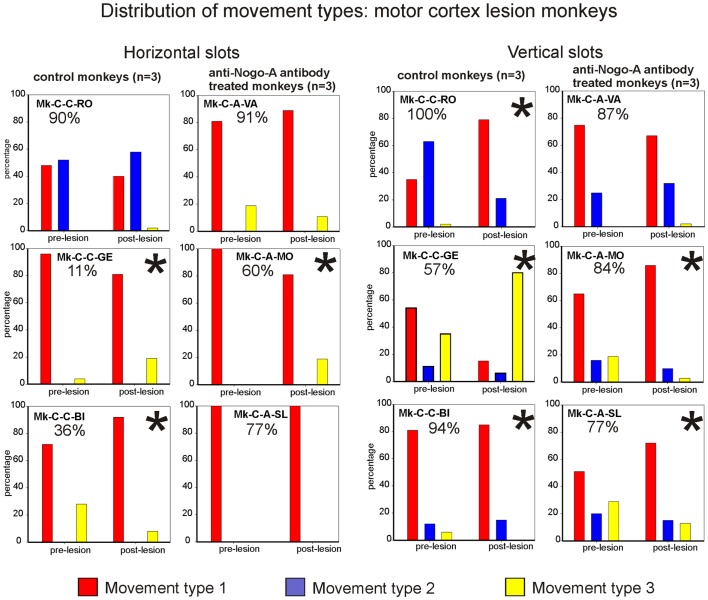
**Distribution of movement types observed for grasping pellets from the horizontal slots (left column) or from the vertical slots (right column) for the six monkeys subjected to motor cortex lesion**. *Significant change in the distribution of movement types for the execution of the task between the pre- versus post-lesion periods (chi-square test, *p* < 0.05).

The analysis of movement strategies showed a statistically significant change in movement type distribution in three monkeys: Mk-C-C-GE, Mk-C-C-BI, and Mk-C-A-MO (Figure 7, left column). It is interesting to note that these monkeys are those characterized by the poorest recovery for the horizontal slots (11, 36, and 60%, respectively). The three monkeys with better functional recovery (above 77%) did not change the distribution of movement type (Figure 7, left column).

##### Results from analysis of movements in cortical lesion monkeys for vertical slots

In monkeys subjected to cortical lesion, and for the vertical slots, statistically significant changes in movement types’ distribution were found in all monkeys, except for Mk-C-A-VA (Figure 7, right column). If one analyses more specifically how the use of the different types of movements varied in the pre- versus post-lesion phase, one can see that four out of six monkeys presented a proportional variation, going toward a stable or more frequent use of the *vertical movement type VMT1*. One can also observe that Mk-C-C-GE rarely using *vertical movement type VMT1* post-lesion is the monkey presenting the worst post-lesion score for vertical slots and that all other monkeys did recover remarkably well (associated to a prominent use of *vertical movement type VMT1*).

#### Summary of the main behavioral results

Overall, the present behavioral data suggest that there is a relationship between the degree of functional recovery from SCI or from motor cortex lesion and the degree of preservation of pre-lesion movement types after functional recovery. In the SCI monkeys, moderate functional recovery (<60%) was associated with a significant change (decrease) of angle of wrist adduction for horizontal slots (Table 2, upper part), as well as a significant change in movement type distribution for vertical slots, with fewer *vertical movement type VMT1* (Figure 6, right column). This pattern corresponds to “functional compensation,” using movement synergies which differ from the pre-lesion pattern. Conversely, better functional recovery (>75%) was most often associated with the restitution of the original pre-lesion distribution of movement types, thus corresponding to a more “true” functional recovery. As far as the motor cortex lesions are concerned, there was no obvious relationship between a change of wrist angle to grasp the pellet post-lesion and the extent of functional recovery (Table 2, lower part). Nevertheless, for the horizontal slots, a change in movement types distribution post-lesion (as compared to pre-lesion) was associated with moderate functional recovery (≤60%), whereas better functional recovery (>77%) occurred when the original distribution of movement types was preserved (Figure 7, left column). Such a relationship was not observed for the vertical slots.

### Pharmacological investigations in motor cortex lesion monkeys

In the six monkeys subjected to motor cortex lesion, the presence of a chronic chamber above the lesioned motor cortex allowed us to perform systematic electrophysiological investigations (ICMS pre- and post-lesion), as well as pharmacological reversible inactivation experiments post-lesion (except Mk-C-C-GE for the latter). In addition, two monkeys derived from a pilot study (32) were added to the pool of monkeys, both subjected to a lesion of the motor cortex, without treatment, referred to as control monkeys Mk-C-C-CE and Mk-C-C-JU (Table 3). The reversible inactivation investigations were aimed at identifying the cortical regions involved in the functional recovery from motor cortex lesion.

**Table 3:** **Survey of the monkeys involved in the reversible inactivation experiments, with indication of the number of sites infused with muscimol for each monkey and each daily session (corresponding to the inactivation of a given motor cortical area)**.

Motor cortical area inactivated	Control monkeys				Anti-Nogo-A antibody treated monkeys		
	Mk-C-C-BI	Mk-C-C-CE*	Mk-C-C-RO	Mk-C-C-JU*	Mk-C-A-MO	Mk-C-A-VA	Mk-C-A-SL^&^
**NUMBER OF INFUSION SITES WITH MUSCIMOL**							
M1	12	9	10	6	20	11	11
PMd	6	8	7	–	7	5	10
PMv	5	8	6	–	4	4	–
PMv/PMd	–	–	–	16	–	–	–
**TOTAL VOLUME OF MUSCIMOL INJECTED (μl)**							
M1	12	9	10	6	20	11	11
PMd	7	8	7	–	7	7.5	10
PMv	5.4	8	6	–	4.4	4.4	–
PMv/PMd	–	–	–	16	–	–	–

*Two pilot monkeys taken from Ref. (32).

*^&^Only the contralesional hand was tested in Mk-C-A-SL*.

*In Mk-C-C-JU, muscimol was infused simultaneously in PMd and PMv*.

*The infusion of muscimol at each site took roughly 3 min (1 min to inject and then 2 min of pause before moving to the next site). As a result, the duration of the infusion procedure ranged between 20 and 40 min*.

*In Mk-C-A-SL, a third inactivation session took place, in which muscimol was infused in M1, targeting a few ICMS sites representing digits in the transition zone toward PMd (referred to as “M1 penumbra” session). A total volume of 9 μl muscimol was infused at nine sites along nine syringe penetrations*.

**Figure 8:**
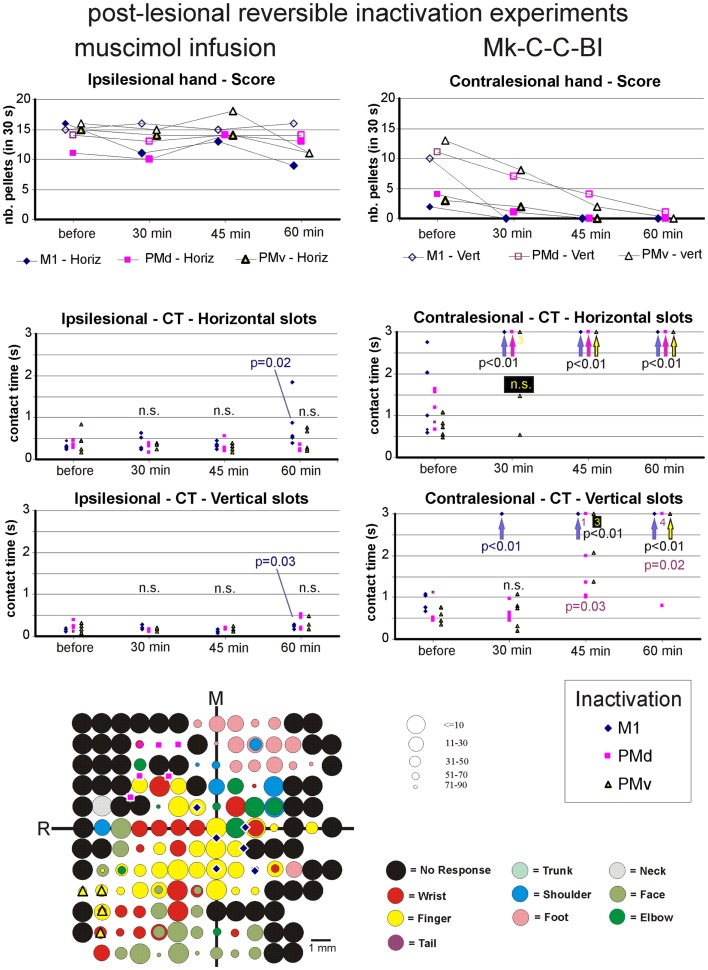
**The top two graphs are plots of the manual dexterity score (number of pellets retrieved in 30 s) as a function of time, namely the four repetitions of the modified Brinkman board task, performed with the ipsilesional hand (left) or the contralesional hand (right) in a given inactivation session (M1, PMd, or PMv)**. The four time points are: before infusion of muscimol, 30 min after (infusion of muscimol), 45 min after, and 60 min after. The scores are shown separately for the vertical (*Vert*) slots and for the horizontal (*Horiz*) slots, as well as for the three sessions in which M1, PMd, or PMv were inactivated, respectively (six different symbols, as coded below the two graphs). As the score is a unique observation at each time point, no statistical comparison could be conducted. The four graphs in the middle are plots of the contact time (in s) measured from the five first horizontal slots (middle two graphs) and from the five first vertical slots (bottom two graphs) attempted by the monkey in the three sessions (inactivation of M1, PMd, or PMv, as shown by the symbols in the bottom right inset). The data for the ipsilesional hand are in the left column, whereas those for the contralesional hand are in the right column. Data points saturated at 3 s are for contact times of at least 3 s or infinitely long (when the monkey was unable to grasp the pellet from the aimed slot). Vertical arrows below the data points at 3 s indicate that all five first attempts were unsuccessful (or took more than 3 s): in such a case, the difference compared to Reference performance (before infusion of muscimol) was highly statistically significant (*p* < 0.01; Mann and Whitney test). In some cases, numbers below the data points saturated at 3 s indicate how many observations were indeed equal to or longer than 3 s. At each time point and for each inactivation session (M1, PMd, or PMv: see color code in the bottom inset), there are five data points when the monkey performed the task successfully (in some cases, two data points may overlap). The contact time values at each of the three time points after infusion of the muscimol (30, 45, and 60 min) were statistically compared with those obtained before infusion of muscimol (reference manual performance). Using the same color code as the symbols, when the difference was statistically significant, the corresponding p value is indicated, whereas “n.s.” means that the difference was not statistically significant (*p* > 0.05). When a single *p* value or “n.s.” is indicated, it means that it holds true for the three inactivation sessions (M1, PMd, or PMv). At the bottom left, the unfolded ICMS map established post-lesion is shown, as it represented the basis to identify positions to perform syringe penetrations to infuse muscimol in M1, PMd, or PMv (same symbols as in the inset on the right). Along the axes, “R” is for rostral and “M” is for medial. In all monkeys (except Mk-C-C-JU), the M1 permanent lesion was in the left hemisphere and therefore the sites of muscimol infusion were ipsilesional in M1, PMd, or PMv. For Mk-C-C-JU, in which the M1 lesion was in the right hemisphere as well as the infusion sites of muscimol, the ICMS map was flipped so that it appears as a left hemisphere map, for better comparison with the other monkeys. The circles represent the positions of electrodes penetrations for ICMS, with color code to represent the body territory activated at the lowest threshold along the corresponding penetration. The threshold value is given by the size of the circle in microAmps (see the right of the ICMS map). The blue diamonds are for the site of infusion of muscimol in M1, the violet squares for the sites of infusion in PMd, and the yellow triangles for the sites of infusion in PMv. The choice of the infusion sites was also based on a confrontation with the unfolded pre-lesion map (not shown). The diamond, square, and triangle symbols show the position of the penetrations with the syringe. Note however that the number of infusion sites may be bigger, as in some individual penetrations muscimol was infused at more than one depth. See Table 3 for the total number of infusion sites and the total volume of muscimol injected.

**Figure 9:**
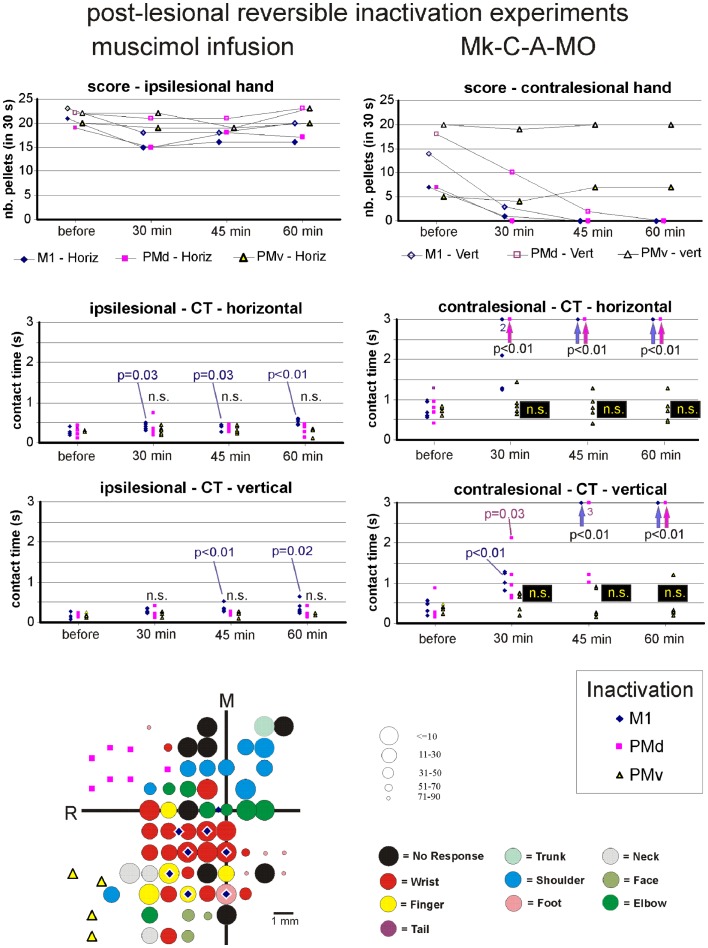
**Inactivation data and post-lesion ICMS data for Mk-C-A-MO (same conventions as in Figure 8)**.

**Figure 10:**
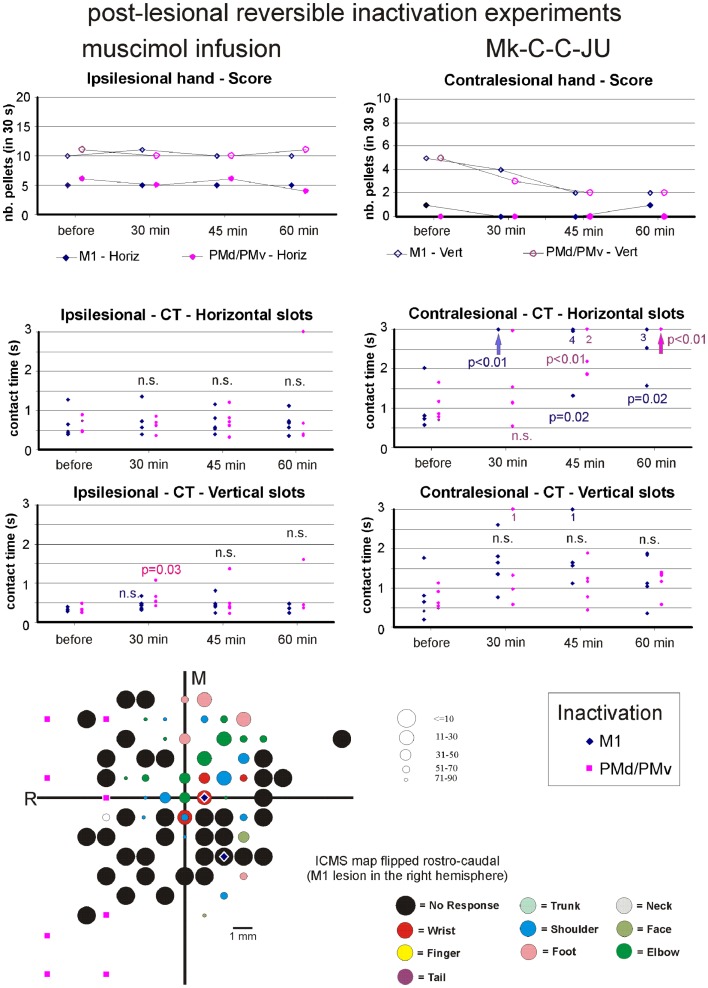
**Inactivation data and post-lesion ICMS data for Mk-C-C-JU (same conventions as in Figure 8)**.

A reversible inactivation session represents a clearly more challenging experiment than a standard behavioral session performed by the monkeys every day during the whole experimental protocol. For that reason, reversible inactivation experiments were scheduled near the end of the experimental protocol, after the monkey reached a stable post-lesion behavioral plateau and after post-lesion ICMS mapping. The reversible inactivation of a given motor cortical area was performed via multiple syringe penetrations to deliver muscimol (see Table 3). To restrict possible tissue damage, only a single reversible inactivation experiment was conducted for each of the three motor cortical areas accessible via the chronic chamber, namely M1, PMd, or PMv (Table 3). In one animal (Mk-C-C-JU), only two reversible inactivation sessions were conducted, one to inactivate M1 and the second aimed at PMd and PMv together (Table 3). In one monkey (Mk-C-A-SL), in contrast to the other animals, only the contralesional hand was tested, due to decreased motivation of the animal at that step of the protocol, prompting for a reduction in the duration of the session by skipping the ipsilesional hand assessment. The data derived from the seven monkeys subjected to reversible inactivation sessions (usually three sessions per animal) are shown in Figures 8–10 for three representative monkeys, where the score and the CT were plotted for comparison between the manual performance before infusion of muscimol, and at three time points following muscimol infusion (30, 45, and 60 min).

For each monkey, several positions on the brain surface were selected for muscimol infusion, based on the post-lesion raw ICMS maps established when the post-lesion plateau was reached. The post-lesion raw ICMS maps used for such selection are displayed for each monkey at the bottom left of Figures 8–10. In some monkeys, there was preservation in M1 after the permanent lesion of a few hand territories (e.g., Mk-C-C-BI, Mk-C-C-RO, Mk-C-A-MO, Mk-C-A-VA, and Mk-C-A-SL); these specific zones were targeted for muscimol infusion. In other monkeys (Mk-C-C-CE, Mk-C-C-JU), although there was no hand representation left, in some ICMS penetrations hand effects were observed at higher intensities (not shown in the ICMS maps), where muscimol was infused. The locations selected for penetrations with the syringe were also consistent with zones delineated in the pre-lesion ICMS maps.

The effect of reversible inactivation of the ipsilesional M1 or PM was tested for two parameters of manual dexterity, namely the score and the CT, both established separately for the vertical and horizontal slots (see Materials and Methods). Furthermore, the effect of muscimol infusion was observed for the affected hand (contralesional) and the ipsilesional hand. The latter acted as a control, as it was expected that reversible inactivation of the motor cortex on one hemisphere should not (or less) affect the ipsilateral hand.

#### Score of manual performance

##### Effect of reversible inactivation on the ipsilesional hand (score)

The ipsilesional hand is controlled mainly by the intact hemisphere. As a result, it was hypothesized that infusion of muscimol in the lesioned hemisphere (in M1, PMd, or PMv) should not affect the manual performance expressed by the *score* for the ipsilesional hand (number of pellets retrieved in 30 s). This hypothesis was verified in monkeys Mk-C-C-BI (Figure 9), Mk-C-C-RO (not shown), Mk-C-C-CE (not shown), Mk-C-A-MO (Figure 9), and Mk-C-C-JU (Figure 10), where the score was largely unchanged as a result of reversible inactivation, except for some variability inherent to the behavioral task itself, especially considering the exceptional duration of the session (about twice long than the standard behavioral sessions without pharmacological inactivation). As a consequence, the motivation of the animal was most likely less constant. In Mk-C-A-VA (not shown), there was a trend toward a progressive decrease in score as a function of post-muscimol infusion time for the three motor areas inactivated, albeit to a limited extent. The ipsilesional hand was not tested in the seventh monkey (Mk-C-A-SL).

##### Effect of reversible inactivation on the contralesional hand (score)

The hypothesis for the contralesional hand is that a transient inactivation of a given motor cortical area (M1, PMd, or PMv) leads to a decrease in the recovered motor performance after permanent lesion of M1 expressed by the *score*, if the corresponding motor cortical area is contributing to the functional recovery of manual dexterity. This hypothesis of a decrease in score was largely verified for the three inactivated motor cortical areas (M1, PMd, or PMv) in Mk-C-C-BI (Figure 8, top right panel), whereas in monkeys Mk-C-A-MO (Figure 9, top right panel), Mk-C-A-VA, and Mk-C-A-SL, the decrease in score was observed after inactivation of M1 or PMd, but not PMv. In Mk-C-C-RO, the effect was limited to the horizontal slots, after inactivation of M1 or PMd, but not PMv. Finally, in monkeys Mk-C-C-JU (Figure 10, top right panel) and Mk-C-C-CE subjected to the largest lesions, there was also a trend toward a decreased score, but which was less obvious due to low reference scores before infusion of muscimol.

#### Contact time

As mentioned above, a clear limitation of the score data is that a single observation was obtained at each time point, preventing statistical analysis. In contrast, the CT based on the grasping duration in five vertical and five horizontal slots is more meaningful as it allows a statistical evaluation, as described below.

##### Effect of reversible inactivation on the contact time for the ipsilesional hand

In line with the hypothesis that the ipsilesional hand is not affected by reversible inactivation of M1, PMd, or PMv, in most cases the CT was not different at various time points after muscimol infusion, as compared to the reference CT before infusion (e.g., middle and bottom left column graphs in Figures 8–10). In a few cases, there was a statistically significant difference, although a closer visual inspection of the plots shows that the clusters of data points are nevertheless not so remote from each other. The episodic differences found for the ipsilesional hand may rather be related to the reversible inactivation experiment imposing exceptionally long daily sessions, thus decreasing the motivation of the monkey at time points after the long process of muscimol infusion. Furthermore, the reference CT values obtained at the onset of the daily session before infusion of the muscimol are short and homogeneous, which favors a statistically significant difference with somewhat more variable data points obtained after reversible inactivation (see for instance Mk-C-C-BI in Figure 8 and Mk-C-A-MO in Figure 9). In spite of these few statistically significant differences the CT data are generally consistent with the score data, indicating an absence (or limited) effect of reversible inactivation of M1, PMd, or PMv on the CT for the ipsilesional hand. Indeed, small volumes of muscimol infused (e.g., 4.4 μl in Mk-C-A-MO and Mk-C-A-VA; see Table 3) may have yielded a statistically significant difference, although the post-infusion manual performance with the ipsilesional hand was excellent (Figure 9).

##### Effect of reversible inactivation on the contact time for the contralesional hand

In contrast to the modest effect on the ipsilesional hand, the reversible inactivation of M1, PMd, or PMv had generally more dramatic effects (and higher levels of statistical significance) on the contralesional hand (Figures 8–10, middle and bottom right column graphs). In Mk-C-C-BI (Figure 8), in line with the score data, the CT for the contralesional hand increased substantially (with high statistical significance) 45 and 60 min after reversible inactivation of all three motor areas (M1, PMd, or PMv). The effect was already largely present 30 min post-infusion. Also consistent with the score data, Mk-C-A-MO (Figure 9) and Mk-VA (not shown) exhibited clear increases in CT for the contralesional hand after inactivation of M1 and PMd, but less so after muscimol infusion in PMv. In Mk-C-C-RO (not shown), the CT obtained for the horizontal slots also showed a strong effect on the contralesional hand of the inactivation of M1 and PMd; the effect was clearly less pronounced for the vertical slots and limited to inactivation of M1. These four monkeys clearly support the notion that ipsilesional motor cortical areas (mainly M1 and PMd) play a role in the incomplete functional recovery of manual dexterity with the contralesional hand after unilateral permanent lesion of M1.

The CT data appear somewhat less straightforward to interpret in the other three monkeys. Unexpectedly, Mk-C-A-SL exhibited an effect of reversible inactivation of M1 or PMd on the contralesional hand for the vertical but not the horizontal slots (not shown). This observation may be explained, at least in part, by the relatively large difficulty the animal already had in performing the grasping from the horizontal slot before reversible inactivation. The results are opposite in Mk-C-C-CE (not shown), in which the effect of the reversible inactivation was prominent (and generally significant) for the horizontal slots and much less for the vertical slots (except maybe 30 min after infusion of muscimol). In Mk-C-C-CE, the increase in CT for the horizontal slots was found after reversible inactivation of all three motor cortical areas (M1, PMd, or PMv), with the exception of M1 after 60 min. In Mk-C-C-JU (Figure 10), subjected to reversible inactivation of M1 or PMd/PMv together, there was also an increase in CT (statistically significant in most cases) for the horizontal slots, but not for the vertical.

### Summary of the main pharmacological results

In conclusion, in spite of some variability inherent to such challenging reversible inactivation experiments (long daily session for the monkeys including penetrations with the syringe in the cerebral cortex), the data are generally consistent with the notion that M1 comprising the permanent lesioned territory and the peri-lesional area, as well as PMd play a crucial role in the functional recovery of manual dexterity following unilateral lesion of the hand area in M1. PMv may also contribute, but to a lesser extent and less systematically than PMd. The effects of muscimol inactivation of M1, PMd, or PMv, as assessed by the CT data, are summarized in Table 4, together with a reminder of the percentages of recovery (post-lesion plateau compared to pre-lesion plateau) for the horizontal and vertical slots in each individual monkey. It appears that the inactivation experiments with muscimol infusion had a stronger impact on the horizontal slots than on the vertical, in other words the corresponding areas may be more involved in functional recovery for the most difficult task (horizontal slots) than for the less difficult (vertical slots). Moreover, the effects observed in the muscimol inactivation sessions do not appear to be different between the untreated (control) group (spontaneous recovery) and the group of monkeys treated with the anti-Nogo-A antibody, suggesting that the corresponding cortical areas contribute to the functional recovery in a comparable manner, irrespective of the treatment.

**Table 4:** **Survey of the reversible inactivation data for the contralesional hand**.

Contralesional hand only	Motor cortical area inactivated	Control monkeys				Anti-Nogo-A antibody treated monkeys		
		Mk-C-C-BI	Mk-C-C-CE*	Mk-C-C-RO	Mk-C-C-JU*	Mk-C-A-MO	Mk-C-A-VA	Mk-C-A-SL
Effect of muscimol infusion on horiz. slots	M1	Yes	Yes	Yes	Yes	Yes	Yes	No
	PMd	Yes	Yes	Yes	–	Yes	Yes	No
	PMv	Yes	Yes	No	–	No	Y/N	–
	PMv/PMd	–	–	–	Yes	–	–	–
Effect of muscimol infusion on vert. slots	M1	Yes	No	Yes	No	Yes	Yes	Yes
	PMd	Yes	No	No	–	Yes	Yes	Yes
	PMv	Yes	No	No	–	No	Y/N	–
	PMv/PMd	–	–	–	No	–	–	–
Volume M1 lesion (mm^3^)		20.13	112.8	14	63.01	41.8	20	78.2
% Recovery horiz. slots		36	9	90	29	60	91	77
% Recovery vert. slots		94	59	100	46	84	87	77

*The effect of muscimol (Yes or No) after injection in M1, PMd, or PMv on the horizontal slots or on the vertical slots for the contralesional hand are based on the contact time data, reflecting here the general tendencies derived from the time points 15, 30 and 45 min post-infusion. Y/N represents divergent observations at two time points (e.g., Mk-C-A-VA). In Mk-C-C-JU, muscimol was injected at the same time in PMd and PMv*.

*Two pilot monkeys taken from Ref. (32).

## Discussion

### Behavioral data

The analysis of movement patterns in the plateau phase of recovery of manual dexterity revealed a relationship between the degree of restoration of the original distribution of movement types and the extent of functional recovery of manual dexterity. However, this relationship was not systematic as the change of wrist angle to grasp pellets from horizontal slots (reflecting compensatory movements) was present in monkeys exhibiting moderate recovery only after cervical cord lesion, but not after motor cortex lesion (Table 2). A change in the distribution of movement types post-lesion (as compared to pre-lesion), also reflecting compensatory movements, was found in monkeys with moderate functional recovery, but only for the vertical slots after cervical cord lesion (Figure 6) and only for the horizontal slots after motor cortex lesion (Figure 7). Reciprocally, the association of better functional recovery with restoration of original movements (“true recovery”) was supported here by the wrist angle analysis in monkeys subjected to cervical cord lesion, but not motor cortex lesion (Table 2) and by the observation of the restitution post-lesion of the original distribution of movement types, but only for vertical slots after cervical cord lesion and only for the horizontal slots after motor cortex lesion. Nevertheless, although not systematic, these relationships between good functional recovery and restitution of original movements are consistent with the study of Murata et al. (40), reporting better functional recovery from motor cortex lesion when the original precision grip was restored as opposed to alternate strategies. Furthermore, these authors demonstrated that intensive motor training post-lesion favored the restoration of the original precision grip movement, as opposed to untrained monkeys using mainly compensatory motor strategies (40). In the present study, all monkeys were subjected to the same regime of post-lesion training, which can be considered as intermediate [daily behavioral assessment as described in Ref. (19)], rather than intensive training [as in Ref. (40) with forced use of the affected hand; see also (41–43)). In studies with much larger sensorimotor cortex lesions, however, no restitution of “true” functional could be observed (44, 45).

In contrast to previously reported lesion models in non-human primates [(15) for SCI and (17) for motor cortex lesion] in which the functional recovery was complete (post-lesion performance at least as good as that pre-lesion), the large majority of our monkeys did not fully recover, both after SCI or motor cortex lesion [see e.g., Figures 2 and 3; see also (12, 13, 32)]. This discrepancy is most likely due to the properties of the different behavioral tasks used to assess deficit and functional recovery. It may be that pellet retrieval from horizontal slots in our modified Brinkman board task is more challenging than the manual dexterity tasks used by others (15, 17).

The choice of using macaque monkeys for this study has several advantages, but also some limitations. First of all, the organization of their central nervous system (CNS) in general and more specifically of their motor system is similar to the human one, and in contrast to rodents [see e.g., (1, 46)]. CM projections for instance are prominent in monkey, as well as human, but absent or limited in rats or mice. The analysis of dexterous movements, such as the precision grip, is only possible in non-human primates or in humans. The results of studies on monkeys are therefore more likely transferable to humans. Furthermore, secondary effects of a treatment can be detected more accurately in monkeys (due to the proximity with the investigators) than in rodents. On the other hand, considering large cohorts of monkeys for such studies is difficult, if not impossible, mainly for ethical reasons. However, interventions or therapies tested extensively in rodents should be transferred to the monkey model for a proof of principle in primates and for safety reasons.

Importantly, behavioral performance can be compared before and after an intervention, a situation that does not occur in humans. The considerable impact of such pre- versus post-lesion direct comparison in individual monkeys has been demonstrated clearly in a recent report (12). Even with standard procedures, the lesions vary within single individuals, like in humans, making individual recovery curves and correlations with anatomical parameters a requirement and a criterion of high heuristic value, sometimes more so than group comparisons.

A critical point in the methodological design of the present study was the choice of the lesion size. The dilemma lies in finding a balance between creating a lesion large enough to result in a distinct and permanent deficit, but small enough so as not to compromise the animal’s health. We used challenging motor tests, which strongly depend on cortical control and could reveal substantial deficits, even in case of smaller lesions. In the present model, when the monkeys were free to move in their home space, it was nearly impossible for a naïve observer to distinguish an intact monkey from a monkey subjected to SCI or motor cortex lesion, even after only a few days. In contrast, the difference was prominent when the monkeys had to perform dexterous movements like in the Brinkman board task [present study; see also Ref. (12, 13)] or the Brinkman box task (21). More precisely, the retrieval of a pellet from a horizontal slot required a postural adaptation of the arm to the slot. This additional movement, not necessary for the execution of the task with vertical slots, increased its complexity and made it more sensitive for the detection of subtle deficits. In the subgroup of monkeys subjected to cervical cord lesion, two of them were *Macaca mulatta* whereas all other monkeys included in the present study were *Macaca fascicularis*. It should be mentioned here that rhesus monkeys (*Macaca mulatta*) have larger hands and digits (and brain), a difference with respect to *Macaca fascicularis* which may have influenced the present results to some extent.

Functional recovery from SCI was improved by anti-Nogo-A antibody therapy, which enhanced sprouting of CS axons above and below the cervical cord injury [(18); see also (47)], thus probably leading to more contacts of CS fibers with distal motoneurons controlling hand muscles. The time course of such axonal sprouting was actually reflected by changes taking place over a few weeks in the ICMS map in the contralesional M1 (27): immediately after the cervical cord lesion, ICMS in M1 no longer elicited digit movements, which re-appeared a few weeks later, in parallel to the behavioral improvement. In addition, after cervical cord hemi-section affecting the CS tract, there is evidence for a role played at different post-lesion stages by the ipsilesional M1 and by PMv (15). Moreover, compensatory sprouting of CS fibers spared by the lesion, descending ipsilaterally and crossing the midline below the lesion, was shown to play a role in the improvement of motor function after hemi-section at C7 level (16). Functional recovery from SCI depends also on other descending tracts, originating from sub-cortical nuclei [e.g., Ref. (48–52)], some of them being spared by the lesion (mostly the reticulospinal tract). The mechanisms of enhanced functional recovery from motor cortex lesion due to anti-Nogo-A antibody treatment may also comprise sprouting and increased plasticity of other tract systems as the antibody in the cerebrospinal fluid reached not only the spinal cord but also large parts of the brain (53). The capacity to grasp pellets does not depend only on motor control *per se*, but also relies on crucial afferent sensory inputs. In the present study, the afferent inputs from the digits may be important for the functional recovery of manual dexterity [see (54, 55)]. However, the question is whether our cervical hemi-section affects the sensory inputs originating from the digits? In a separate study, in some of our monkeys subjected to cervical cord lesion at C7 level, the tracer cholera-toxin B subunit was injected in the digits in order to trans-ganglionally trace the afferent sensory inputs. The labeling of axon terminals in the cuneate nucleus was comparable on both sides (unpublished data), in spite of a unilateral lesion, suggesting that the afferent inputs from the digits are largely intact and therefore in a position to contribute to the functional recovery. In other words, the deficit in recovery from cervical lesion is mostly motor, with the possibility to benefit from largely preserved somatosensory feedback. In future experiments on monkeys initially subjected to a cervical cord lesion, only a subsequent selective lesion of the afferent inputs may tell the extent to which the sensory inputs contribute to the functional recovery of manual dexterity.

### Pharmacological data

In contrast to intact monkeys in which PMd was reversibly inactivated without an effect on manual dexterity of the contralesional hand (38), the present study demonstrates that reversible inactivation of PMd in monkeys subjected several months earlier to a permanent lesion of the hand representation in M1 indeed provokes a loss of the recovered manual dexterity, irrespective of whether the recovery was spontaneous or enhanced with an anti-Nogo-A antibody treatment. In other words, PMd contributes crucially to the functional recovery from M1 lesion, thus confirming the pilot data of Liu and Rouiller (32), derived from two control (untreated) monkeys (Mk-C-C-JU and MkC-C-CE). A major difference in the analysis was that in the report by Liu and Rouiller (32), the data were restricted to the score (no CT measurement) and the number of pellets was given only by a total value, without distinction between the vertical and horizontal slots (introduced in the present study). Furthermore, the score values in Liu and Rouiller (32) were the number of pellets retrieved in 60 s whereas, in the present study, a period of 30 s was considered, justified by the observation that in some cases after the cortical lesion the monkeys did not work as long as 1 min. The distinction between vertical and horizontal slots is also of considerable importance, due to the different degree of difficulty to grasp the pellet depending on the slot orientation [see e.g., (11, 12); see also (45)].

As compared to the pilot reversible inactivation data of Liu and Rouiller (32), a further major advance of the present study was to introduce the investigation of CT, allowing statistical analysis, yielding pertinent data providing the monkey performs the task for at least five vertical and five horizontal slots, which was usually the case (Figures 8–10). The contact time is also more specific than the score to assess manual dexterity, as the score comprises not only the precision grip phase, but also the transport of the pellet from the board to the mouth and the transport of the empty hand back to the board to aim toward the next slot (see also (15) for use of CT).

In most monkeys newly introduced here (Mk-C-C-BI, Mk-C-C-RO, Mk-C-A-MO, Mk-C-A-VA, and Mk-C-A-SL) and not yet available in the pilot study of Liu and Rouiller (32), the effect of reversible inactivation of the lesioned M1 zone was more systematic and more pronounced (with the exception of Mk.C-A-SL) than in the pilot monkeys Mk-C-C-CE and Mk-C-C-JU. This more prominent effect of inactivating the lesioned M1 in these more recent monkeys (Mk-C-C-BI, Mk-C-C-RO, Mk-C-A-MO, Mk-C-A-VA) than in the pilot monkeys Mk-C-C-CE and Mk-C-C-JU is consistent with the notion that the permanent lesion in M1 is smaller in the recent monkeys than in the two pilot ones. Consequently, in the case of a smaller lesion, there is more preserved adjacent territory in M1 most likely involved in the functional recovery [see (56) for the role played by adjacent territory to a restricted lesion in M1]. When inactivated with muscimol, the effect on the recovered manual dexterity is larger than in animals with less territory in M1 involved in the functional recovery. This interpretation is supported by the case of Mk-C-A-SL, subjected to a large permanent lesion (comparable to that in Mk-C-C-JU): the reversible inactivation of the lesioned M1 provoked a moderate effect limited to the vertical slots, consistent with a less strong effect observed in the other two monkeys with a large permanent lesion of M1 (Mk-C-C-CE and Mk-C-C-JU). In case of a large permanent lesion in M1, the mechanisms of functional recovery are more dependent on non-primary motor cortical areas, such as PM [(32); present study] or SMA (57, 58).

The main conclusion of the present reversible inactivation study is that, after a unilateral permanent lesion of the hand area in M1, peri-lesional M1 territories played a significant and systematic role in the functional recovery of the contralesional hand (Figures 8–10; Table 4). The same was largely true for the ipsilesional PMd, with however a few exceptions for the vertical slots at one or the other time point in the daily testing sessions (Table 4). On the other hand, PMv seemed to play a less prominent role (Table 4): a contribution of PMv to the functional recovery of manual dexterity was found in Mk-C-C-BI (vertical and horizontal slots), in Mk-C-C-CE (horizontal slots only), in Mk-C-A-VA (both slot orientations but at a single time point), but not in monkeys Mk-C-C-RO and Mk-C-A-MO. This observation may be explained by smaller volumes of muscimol injected in PMv (Table 3), because of more restricted access to PMv than PMd via the chronically implanted chambers. Indeed, small volumes of muscimol (4.4 μl) were injected in Mk-C-A-MO and Mk-C-A-VA, in which the inactivation of PMv was ineffective and an effect of PMv inactivation was found in Mk-C-C-CE, in which the volume infused in PMv was as large as that injected in PMd (8 μl; Table 3). However, this interpretation is not straightforward, as shown by the case of Mk-C-C-BI, in which the inactivation of PMv affected the CT in spite of a relatively small volume of muscimol injected (5.4 μl; Table 1). Mk-C-C-RO, in spite of a larger volume of infusion in PMv (6 μl; Table 1), did not exhibit an effect on CT after inactivation of PMv.

As far as the reversible inactivation technique with infusion of muscimol is concerned, the control experiment of injecting saline instead of muscimol has not been conducted in the course of the present study to avoid additional sessions with the risk to damage the cortex with multiple syringe penetrations. Nevertheless, this control experiment has been conducted several times in our laboratory in previous studies on intact monkeys (38, 59): the infusion of saline instead of muscimol in M1, or in SMA or in PM (same volume and same number of sites) did not affect the motor performance of the monkey in the modified Brinkman board task or in the reach and grasp drawer task.

An important issue concerns the possibility that the reversible inactivation with muscimol of PMd or PMv does not inhibit the functional role of PM in the functional recovery *per se* but rather that muscimol may diffuse passively from the injection sites in PM to M1, thus inhibiting M1 in a second step. Again, previous studies conducted in intact monkeys argue against such interpretation. As shown in Kermadi et al. (38), the deficits observed after infusion of muscimol in SMA or PM were clearly different from the deficits resulting from infusion in M1, up to at least 1 h post-injection of muscimol. The post-lesion ICMS maps, illustrating the position of the muscimol infusion sites in PMd or PM, show that the sites in PMd or PMv closest to M1 are located, in most cases, at 2–3 mm from the initial hand representation in M1, the other infusion sites in PMd or PMv being more distant. Based on the estimated distance of diffusion of the inactivating agent [1–2 mm, see (37)], concern about direct diffusion from PM to M1 is most likely minor.

The present reversible inactivation sessions based on infusion of muscimol are challenging experiments, and therefore there are limitations in their interpretation. First, the daily session in which the monkey had to repetitively perform the manual task with each hand was long due to the time needed to infuse the muscimol at several sites. Nevertheless, the data obtained at the two or three time points tested post-infusion were generally comparable, indicating that a drop of motivation of the monkey was not a major concern. Second, due to limitations in the extent of territories accessible from the chronic chamber, PMd and PMv could not be fully characterized in all monkeys (see ICMS maps in Figures 8–10) and therefore the choice of the sites to infuse muscimol was less reliable than in M1.

Based on the pilot data of Liu and Rouiller (32), with evidence for a role played by PM in the functional recovery, the chronic chamber implanted in the subsequent monkeys was aimed to the lesioned M1 and PM. Inactivating other cortical areas such as SMA would have been of interest [see (57, 58, 60), as well as the cingulate motor cortical area (CMA)], plus the motor cortical areas in the intact hemisphere. Such a broader investigation of other cortical areas would require larger and more chronically implanted chambers, with higher risks of compromising the long overall duration of the experiment.

The present reversible inactivation data confirm the role played by the ipsilesional PM (PMd and, to a lesser extent, PMv) in the functional recovery from a permanent lesion of the hand representation in M1, in line with previous reports using a different model of cortical lesion (61–63). A similar role for PM was reported in patients [e.g., (64)]. The question is how PM exerts its influence on the functional recovery of manual dexterity. In intact animals, the influence of PM, especially PMv, on manual dexterity, is exerted in large part via its corticocortical projection to M1 (65, 66). This observation is consistent with the notion that the CS projection originating from PM terminates primarily in cervical segments C3–C4, located more rostrally than the hand motoneurons at spinal levels C8-T2. One cannot, however, exclude that some motor effects from PMv may be exerted via its CS projection, then relayed to lower spinal segments via propriospinal projections [see e.g., (67)]. In the present monkeys subjected to a permanent lesion of M1, the role played by PM (PMd and/or PMv) in the functional recovery of manual dexterity may depend on different pathways. First, in monkeys in which some peri-lesion hand territories in M1 are preserved, PM may enhance its indirect influence via M1, following the mechanisms reported by Schmidlin et al. (65) in intact animals. Second, the influence of PM on functional recovery may be exerted via its CS projection, which may be enhanced in the hypothetic case of sprouting of the axons terminals to reach the more caudal hand motoneurons [see also (58) for a similar mechanism for CS projections originating from the ipsilesional SMA]. Third, as demonstrated anatomically by Dancause et al. (62), after permanent lesion of M1, there is an enhancement of the projection from PM (specifically PMv) to the primary somatosensory cortex (S1), which may improve motor control based on sensorimotor integration. The last two mechanisms may be accentuated by the anti-Nogo-A antibody treatment. Fourth, along the same line, there is evidence in the anti-Nogo-A antibody treated monkeys subjected to M1 lesion for an enhancement of callosal projections reaching the ipsilesional PM (21). Moreover, changes in indirect cortico-bulbar/bulbo-spinal pathways may also contribute to the functional recovery.

### Conclusion

We compared the functional recovery after a unilateral SCI and motor cortex lesion in adult macaque monkeys using a sensitive manual dexterity task (modified Brinkman board) as a test system. The duration of motor deficits was generally comparable for both types of lesion (a few weeks), although variable across monkeys depending on the lesion size and precise position. The effects of the lesion were somewhat more pronounced after *cervical cord lesion* (average functional recovery in the three control monkeys was 59% for the vertical slots and 40% for the horizontal) than after *motor cortex lesion* (average functional recovery in the three control monkeys was 84 and 46% for the vertical and horizontal slots, respectively; see Table 1 for individual data). The expected effects of the two lesion types are different, as the cortical lesion affects a part of the CS projection originating from the M1 hand area, whereas the cervical cord lesion is less specific as it affects the CS projection coming from M1, plus from other cortical areas (e.g., premotor cortex, supplementary motor area, cingulate motor area). This is consistent with the observation of a less extensive functional recovery on average in control monkeys after cervical cord lesion, as compared to motor cortex lesion. However, the difference between the two types of lesion is not so great, as it may be counterbalanced to some extent by the (indirect) impact of the motor cortex lesion on other descending tracts (loss of some cortico-bulbar influences) which is less present in the cervical cord lesion, as long as the damage is restricted to the dorsolateral funiculus (see however Figure 1A showing that a few non-CS tracts are preserved, mainly in the ventral funiculus).

In monkeys with destruction of the M1 hand area, adjacent peri-lesion M1 areas as well as PMd and, to a lesser extent PMv, were involved in this recovery, as shown by temporary inactivation with muscimol. In SCI animals, if some non-CS tracts are preserved in part, one may speculate that M1 may still orchestrate a re-arrangement of influences along sub-cortical descending pathways not affected by the lesion (including plasticity at the level of bulbar and spinal circuits).

The relationship found in the present study between the degree of restitution of original movements and the extent of functional recovery, though not systematic (see Table 2; Figures 5 and 6), adds evidence in favor of the concept (in the clinical context) that rehabilitative training should preferentially aim at restoring original movement patterns, as opposed to strategies of substitution [in line with (40)]. Moreover, future treatments should, in addition to physiotherapy [and possibly combined pharmacological treatments; see Ref. (68)], aim at restoring the original neural circuits as much as possible, thus creating the most favorable conditions for efficient functional recovery.

## Conflict of Interest Statement

The antibodies were provided by Novartis Pharma AG.

## Supplementary Material

The Supplementary Material for this article can be found online at http://www.frontiersin.org/Movement_Disorders/10.3389/fneur.2013.00101/abstract

Six video sequences illustrate the three movement types used by the monkeys to grasp pellets from the horizontal slots (HMT1, HMT2, HMT3) and the three movement types used by the monkeys to grasp pellets from the vertical slots (VMT1, VMT2, VMT3). Same material accessible at: http://www.dartfish.tv/Dispatch.aspx?target=collection&CR=p96281c64853&sh=li&aid=cfe44ff5-27f3-4c48-afaa-f23d08a82919

## Footnotes

^1^A new Swiss regulation has been introduced in September 2010 requesting now a volume of 45 m3 at least to be given to a group of up to five macaque monkeys.
